# Current Progress of Hederagenin and Its Derivatives for Disease Therapy (2017–Present)

**DOI:** 10.3390/molecules30061275

**Published:** 2025-03-12

**Authors:** Wang Wang, Yan Jin, Meng-Ke Liu, Sai-Yang Zhang, Hong Chen, Jian Song

**Affiliations:** 1 Luoyang Key Laboratory of Organic Functional Molecules, School of Food and Drug, Luoyang Normal University, Luoyang 471934, China; wangwang43@lynu.edu.cn (W.W.); 15037744652@163.com (Y.J.); 18236979177@163.com (M.-K.L.); 2 School of Basic Medical Sciences, Zhengzhou University, Zhengzhou 450001, China; saiyangz@zzu.edu.cn; 3 State Key Laboratory of Esophageal Cancer Prevention & Treatment, Zhengzhou 450001, China

**Keywords:** hederagenin, structural modification, bioactivity, drug discovery

## Abstract

Natural products have emerged as crucial sources of biologically active compounds, holding promise for applications in drug development. Among the extensively researched pentacyclic triterpenes, hederagenin (HG) stands out for its diverse biological activities and serves as a valuable scaffold for synthesizing novel derivatives. These derivatives hold significant promise for the development of novel therapeutic agents aimed at treating a wide range of diseases. Over the past years, a multitude of HG derivatives with varied bioactivities have been synthesized through chemical modifications. This review article consolidates the most recent findings (since 2017) on HG derivatives, emphasizing their biological effects and mechanisms of action in both in vitro and in vivo models. The objective of this compilation is to offer insights and direct future research endeavors in the realm of HG.

## 1. Introduction

**Figure 1:**
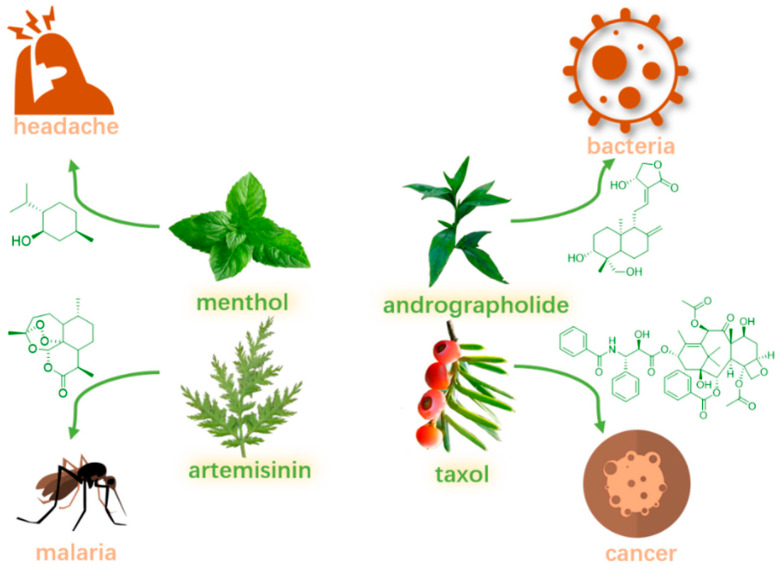
Some terpenes with good clinical effects.

Medicinal herbs and natural products have long been employed in the prevention and treatment of diseases, owing to their abundant sources and potent biological activities [1]. Terpenes, a diverse class of natural compounds abundantly found in medicinal plants, represent a therapeutically significant group of substances, as demonstrated by clinically valuable molecules such as taxol, menthol, andrographolide, and artemisinin (Figure 1) [2,3,4]. Triterpenes, synthesized in plants through the cyclization of squalene (comprising six isoprene units), exhibit a diverse array of bioactivities. Extensive research has documented their anti-cancer [5], anti-inflammatory [6], anti-viral [7], anti-diabetic [8], anti-microbial [9], cardioprotective [10], hepatoprotective, and gastroprotective effects [11,12].

**Figure 2:**
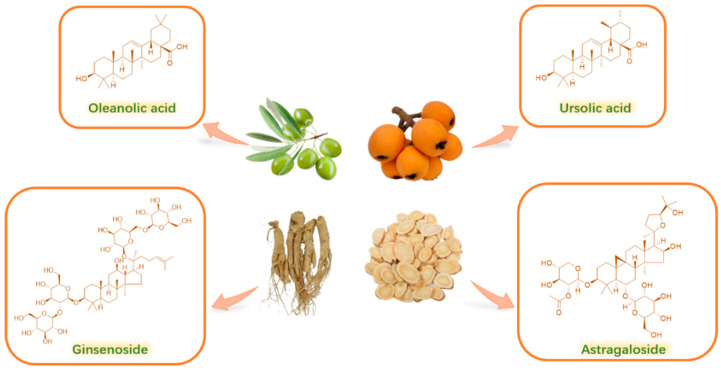
Some triterpenes with fine clinical effects.

In China, some triterpenes have already been employed in clinical settings for the therapy of liver diseases (oleanolic acid) [13], cardiovascular diseases (astragalosides) [14], or as health care products (e.g., ursolic acid and ginsenoside) [15,16], which exhibit good therapeutic effects (Figure 2). Triterpenes are abundant in plants and easy to extract. It is well worth finding and developing a triterpene drug with enormous medical and economic value.

Recently, hederagenin (HG), a pentacyclic triterpene, has attracted significant attention from researchers. Numerous studies have demonstrated that HG and its derivatives possess diverse biological activities, including anti-cancer [17], anti-inflammatory [18], anti-leishmanial [19], anti-diabetic [20], and anti-depressant effects [21], among others [22]. Furthermore, HG serves as a promising scaffold for developing new derivatives with enhanced bioactivity, bioavailability, and pharmacokinetic profiles. In a comprehensive review, Zeng et al. summarized the research and development of HG over the past 50 years up to 2017 [22]. Capitalizing on these advancements, researchers worldwide have increasingly focused on the structural modification of HG and the exploration of its associated biological activities in recent years. In this paper, we classify and summarize the recent advancements in the drug chemistry and pharmacology of HG, based on relevant biological and pharmacological studies conducted from 2017 to the present. The aim of this review is to underscore the therapeutic promise and significance of HG as a model for the development of novel drugs.

## 2. Properties and Sources

HG, a biologically active pentacyclic triterpene of the oleanane type, is widely distributed in various edible and medicinal plants. This compound derives its name from its particularly high concentration in the seeds of English ivy (*Hedera helix*). Additionally, HG can be readily extracted from the fruit of *Fructus Akebiae* (commonly known as ’August bomb’ in China) and the leaves of *Cyclocarya paliurus* (a traditional Chinese herb also called ‘sweet tea tree’) [23]. In plants, HG exists primarily as a free acid but can also be conjugated with one or more sugar units to form glycosides, a characteristic shared with other triterpenes.

In its pure form, HG exists as a white crystalline powder with a characteristic bitter taste. Similar to most triterpenoids, it exhibits poor solubility characteristics, demonstrating limited dissolution in both aqueous and alcoholic solutions. However, it shows appreciable solubility in polar aprotic solvents such as dimethyl sulfoxide (DMSO) and dimethylformamide (DMF). Because HG is insoluble, its bioavailability and absorption are low in vivo, potentially restricting its clinical uses. HG is an aglycone (without sugar) that is present in other molecules that have sugar units bonded to their structural elements. Through hydrolysis to remove the sugar molecules or digestion, these glycosides could be transformed in vivo into HG [24].

## 3. Sites of Modification

**Figure 3:**
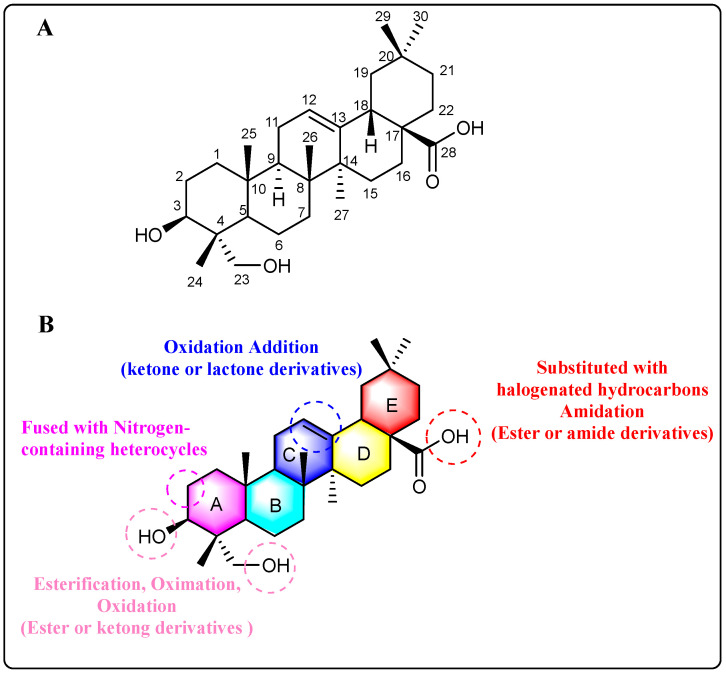
(**A**) The chemical structure of HG. (**B**) The main sites of structural modification and corresponding methods of HG.

The structural framework of HG shares significant homology with oleanolic acid, differing primarily in the presence of a C-23 hydroxyl group, as depicted in Figure 3A. The most reactive sites for structural modification are the hydroxyl groups at C-3 and C-23 positions, along with the carboxyl group at C-28. The reactivity of C-28 carboxyl group is lower compared to typical carboxyl groups; it can undergo amidation with amines and substitution by halogenated hydrocarbons but cannot be esterified with alcohols. In contrast, C-3 and C-23 hydroxyl groups can be esterified with carboxylic acids or oxidized to carbonyls. The A and B rings of HG share structural similarities with andrographolide. When the C-3 hydroxyl group is oxidized, HG can undergo a condensation reaction with aldehydes at the C-2 position. This reaction allows for the fusion of nitrogen-containing heterocycles to the A-ring through condensation. Additionally, the A-ring can be transformed into caprolactam via the Beckmann rearrangement [25].

Recent studies have demonstrated growing interest in structural modifications of HG’s C-ring, with particular emphasis on the C-11,12 double bond. Such modifications have shown promising potential in modulating cellular signaling pathways and inducing various biologically relevant responses in vivo. Nevertheless, the inherent chemical reactivity of this conjugated double bond presents significant synthetic challenges and may contribute to off-target effects in biological systems. Figure 3B provides a concise summary of the primary structural modification sites and corresponding methods employed in HG modifications.

## 4. Derivatives and Bioactivities

HG exhibits a broad spectrum of biological activities, with anti-cancer effects being the most extensively studied. Additionally, its anti-inflammatory [18,26], anti-depressant [27,28], anti-neurodegenerative [29,30], anti-hyperlipidemia [31], anti-diabetic [32], and anti-leishmanicidal [19] activities have also garnered significant attention. However, some of these bioactivities require further in vivo validation, and the underlying mechanisms of action necessitate deeper investigation. In this section, we systematically summarize the primary biological activities and the corresponding mechanisms of HG and its derivatives.

### 4.1. Anti-Cancer Activity

**Figure 4:**
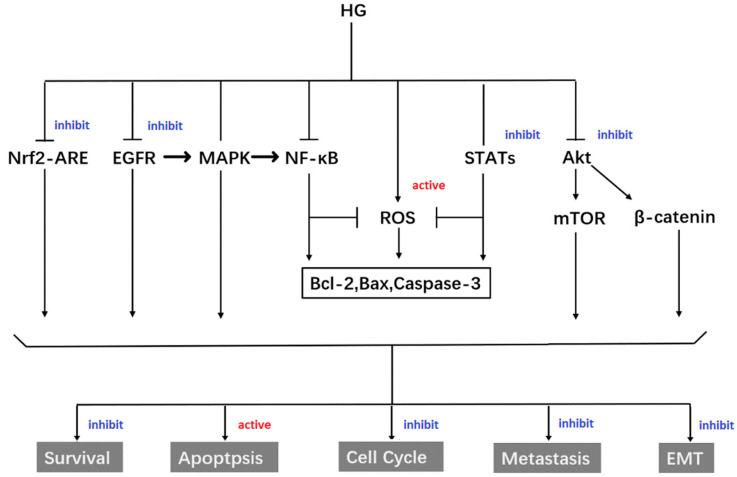
Mechanism underlying the anti-cancer effect of HG.

A multitude of studies have demonstrated the significant anti-cancer efficacy of HG against more than 50 types of cancer cells. Zeng’s comprehensive review summarizes the cytotoxic activity of HG reported during the pertinent period [22]. Extensive research has been conducted to elucidate the anti-cancer mechanisms of HG, including the inhibition of nuclear factor kappa B (NF-κB), modulation of kinases [33], and induction of the PI3K/AKT signaling pathway (as illustrated in Figure 4) [34]. To further enhance the anti-cancer activity and solubility of HG, researchers are actively synthesizing derivatives using various methodologies.

As a byproduct of oxygen metabolism in living cells, reactive oxygen species (ROS) play a pivotal role in maintaining cellular homeostasis. Accumulating research evidence indicates that various natural compounds could influence apoptosis or the cell cycle in cancer cells by elevating ROS levels [35,36,37]. In 2017, Kim et al. demonstrated that HG selectively induces apoptosis in head and neck cancer (HNC) cells through mitochondrial membrane potential (ΔΨm) modulation and subsequent activation of apoptotic pathways. Their mechanistic studies revealed that HG exerts its anti-cancer effects by dual regulation of the Nrf2-ARE antioxidant pathway and p53 tumor suppressor, leading to elevated ROS levels and accelerated glutathione depletion in HNC cells. This study was the first to report that HG can induce cancer cell death through modulation of the Nrf2-ARE pathway [38]. Additionally, Wang et al. observed that HG increases autophagosome formation in NCI-H1299 lung cancer cells, accompanied by upregulation of LC3-II and p62, while also inhibiting lysosomal acidification. Furthermore, HG potentiates the pro-apoptotic effects of cisplatin and paclitaxel by accumulating ROS, thereby enhancing their therapeutic efficacy [39].

An increase in ROS may serve as a prerequisite for potential disruption of the mitochondrial membrane, ultimately inducing cell apoptosis [40,41]. Liu et al. demonstrated that HG treatment induced G2/M phase cell cycle arrest in HepG2 cells, accompanied by significant alterations in the expression profile of cell cycle regulatory proteins. Through fluorescence microscopy analysis, the researchers further revealed that HG treatment caused substantial mitochondrial dysfunction in HepG2 cells, as evidenced by a marked reduction in mitochondrial mass. Further analysis revealed that HG decreased ΔΨm and led to the cleavage of cytochrome C and activation of caspase-9, ultimately triggering the apoptotic process [42].

HG exerts anti-cancer effects by regulating the activation and inhibition of various kinases and their downstream signaling cascades via phosphorylation and dephosphorylation mechanisms. Dai et al. observed that HG significantly downregulated the expression of Nur77, PI3K, and AKT genes, along with reducing the protein levels of Nur77, phosphorylated PI3K, and phosphorylated AKT. These results suggest that HG exerts its anti-cancer effects on glioma cell biological activities through modulation of the Nur77/PI3K/AKT signal pathway [43].

Unlike small molecule drugs, natural products typically exhibit pleiotropic effects by targeting multiple proteins, which significantly complicates the identification of their precise cellular targets [44]. However, advancements in methodologies have facilitated this process in recent years. Zhang et al. employed an advanced quantitative proteomics approach, utilizing tandem mass tag (TMT) labeling coupled with liquid chromatography-tandem mass spectrometry (LC-MS/MS), to systematically analyze differentially expressed proteins in human glioblastoma U87 cells in response to HG treatment. Quantitative protein profiling identified a total of 6522 proteins, with WB analysis revealing significant downregulation of KIF7 and ATAD2B and upregulation of PHEX and TIMM9. Notably, the inhibition of GBM-U87 cells by HG may be linked to KIF7 and the Hedgehog signaling pathway [45]. Furthermore, Su et al. demonstrated that HG alters mitochondrial morphology by suppressing dynamin-related protein 1 (Drp1), a vital mitochondrial fission factor, using MitoTracker Red staining, flow cytometric analysis, and Western blotting. They found that overexpressing Drp1 reversed HG-induced apoptosis, while knocking down Drp1 exacerbated it. Additionally, HG has been shown to trigger mitochondrial BAX translocation and induce apoptosis in ovarian cancer cells [46].

Ferroptosis, an iron-dependent form of cell death associated with lipid peroxidation, has emerged as a promising therapeutic approach for cancer treatment [47]. Lu et al. investigated the role of HG in inducing ferroptosis by examining the expression of CHAC1, a known ferroptosis-inducing gene, through apoptosis gene detection, bioinformatics analysis, and RT-qPCR [48]. The researchers employed both loss-of-function and gain-of-function approaches to modulate CHAC1 expression in lung cancer cells, utilizing RNA interference for gene knockdown and plasmid transfection for overexpression. Functional assays revealed that CHAC1 upregulation significantly enhanced cancer cell death, as demonstrated through comprehensive phenotypic characterization. Functional rescue experiments and in vivo studies further confirmed that downregulation of CHAC1 reversed the HG-induced promotion of lung cancer cell death. Collectively, these findings suggest that HG exerts an anti-cancer effect by promoting CHAC1-mediated ferroptosis.

**Figure 5:**
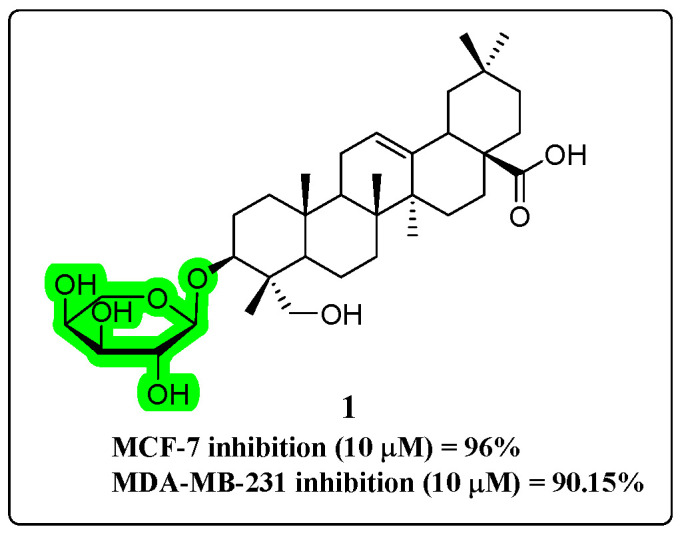
The chemical structure of compound **1** as an anti-cancer derivative.

Clematis hederagenin saponin (compound **1**, Figure 5) is a monoglycoside derivative of HG that elicits cytotoxic effects in breast cancer cells. Furthermore, it exhibits the capacity to induce apoptosis in these cells over an extended period. Mechanistic studies revealed that compound **1** significantly reduces the levels of mitochondrial Apaf-1 and cytochrome C proteins while concurrently enhancing the activities of caspase-3 and caspase-9 [49].

**Figure 6:**
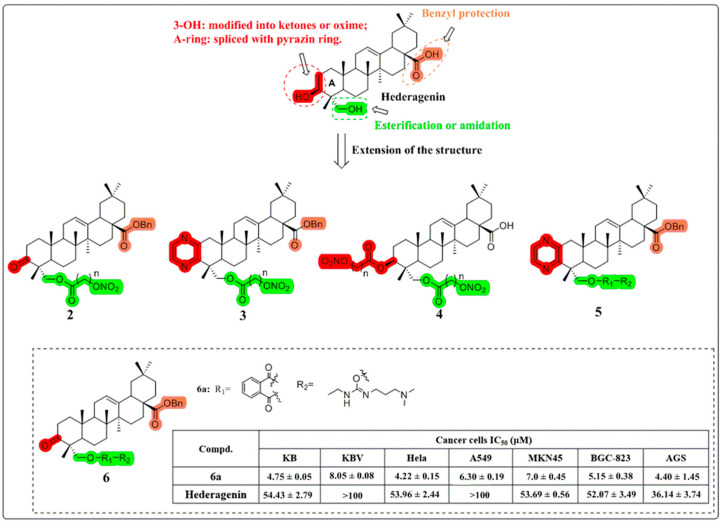
The chemical structures of compounds **2**–**6** as anti-cancer derivatives.

Yi Bi et al. focused on the synthesis of HG derivatives and achieved many successes [50]. In 2017, they synthesized 24 HG derivatives using a diverse modification site strategy (Figure 6) and evaluated them for in vitro cytotoxicity. The polyamine derivative **6a** showed higher potency than HG against KB cells. It increased the Bax/bcl-2 ratio, which disrupted mitochondrial potential and induced apoptosis.

**Figure 7:**
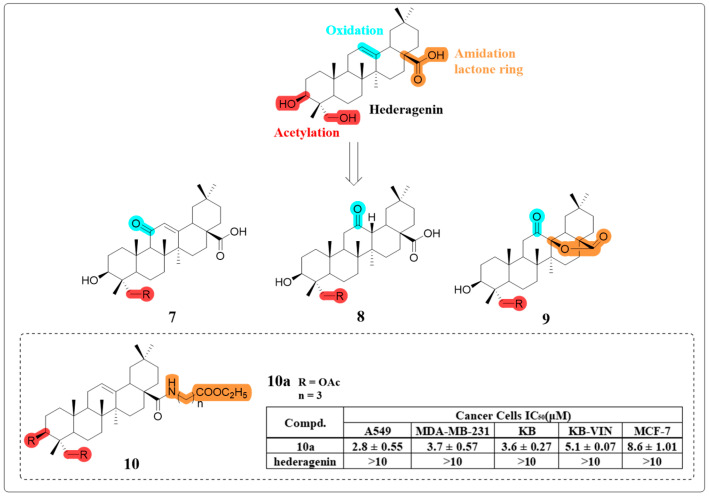
The chemical structures of compounds **7**–**10** as anti-cancer derivatives.

Certain HG glycosides possess robust anti-cancer activities, yet their clinical progression has been hindered by significant hemolytic toxicity [51]. In a study led by Kuo-Hsiung Lee’s group, seventeen HG derivatives were synthesized with modifications at several positions (Figure 7). These derivatives were then assessed for their cytotoxicity against multiple human cancer cell lines and for hemolytic toxicity against rabbit erythrocytes. Compound **10a** demonstrated potent cytotoxic activity against human A549 non-small cell lung cancer cells, exhibiting a dose-dependent response with an IC_50_ value of 2.8 μM, while maintaining excellent hemocompatibility. Mechanistic investigations revealed that compound **10a** exerted its anti-cancer effects through dual induction of G1 phase cell cycle arrest and apoptosis, mediated by simultaneous activation of both intrinsic and extrinsic apoptotic pathways [52]. The SAR study showed that the modifications of oxidation and lactone ring formation on the C ring have no significant impact on the anti-cancer activity. The amidation and esterification of the C-28 carboxyl group can be implemented easily and in high yield, and the product usually has improved activity compared to HG.

**Figure 8:**
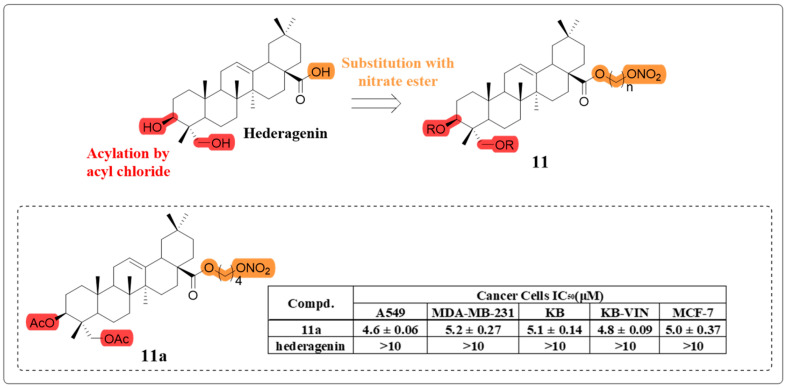
The chemical structure of compound **11** as an anti-cancer derivative.

Nitric oxide (NO) serves as a crucial signaling molecule, mediating both intracellular and intercellular communication, and regulates diverse physiological processes across multiple biological systems. At elevated concentrations, NO generated from pharmacological donors has been demonstrated to exert potent anti-cancer effects, including induction of apoptotic cell death, suppression of metastatic potential, and sensitization of malignant cells to conventional therapeutic interventions [53]. Building upon previous research, Lee’s group synthesized a series of hybrid molecules the following year, incorporating a nitrate ester-based NO donor moiety at the C-28 carboxyl group of HG (Figure 8). Among these, compound **11a** exhibited the most potent antiproliferative activity and compared to HG, compound **11a** demonstrated a more pronounced inhibitory effect on EGFR-LTC kinase activity. Importantly, it generated the highest levels of NO in H1975 cancer cells, suggesting a potential synergistic role of NO in its anti-cancer effects [54].

**Figure 9:**
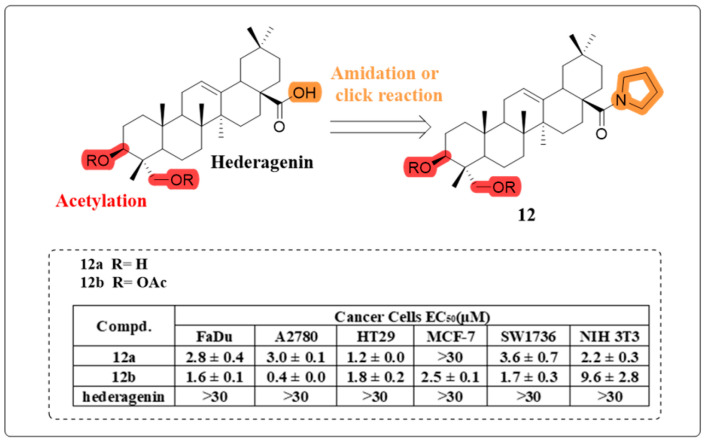
The chemical structure of compound **12** as an anti-cancer derivative.

The research group led by Rene Csuk successfully developed a series of C-28 amide derivatives of HG incorporating nitrogen-containing heterocyclic structures. Biological evaluation revealed that most of these synthesized amide derivatives displayed potent cytotoxic effects against a panel of human cancer cell lines. Particularly noteworthy was the observation that acetylated derivatives consistently exhibited superior anti-cancer activity when compared to their hydroxylated analogues. Among the hydroxylated derivatives, **12a**, which featured a pyrrolidinyl substituent, exhibited the greatest potency against HT29 human cancer cells. However, the acetylated derivative, **12b**, demonstrated the highest efficacy and selectivity against A2780, FaDu, and SW1736 cells (Figure 9). Furthermore, docking studies revealed that acetylated compounds exhibited a stronger affinity for HER2 than for USP7, suggesting HER2 as the likely receptor target, given its presence alongside USP7 in the A2780 cancer cell line [55].

**Figure 10:**
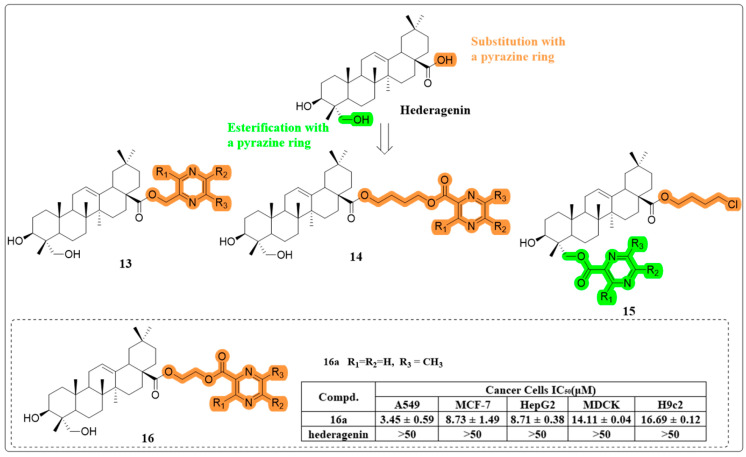
The chemical structures of compounds **13**–**16** as anti-cancer derivatives.

In their quest for more effective anti-cancer agents, Fang et al. synthesized four series of HG-pyrazine derivatives, with the pyrazine moiety esterified either with a C-28 carboxyl group or a C-23 hydroxyl group (Figure 10). The majority of these derivatives exhibited significantly heightened cytotoxic activity compared to HG. Of particular interest, compound **16a** demonstrated the highest potency, inducing early apoptosis and cell cycle arrest in the S phase in A549 cells. A subsequent structure–activity relationship (SAR) analysis reinforced the crucial role of pyrazine in potentiating the anti-cancer effect of HG [56].

### 4.2. Anti-Fungal and Anti-Leishmania Activity

Pneumolysin (PLY), a crucial virulence determinant of Streptococcus pneumoniae, possesses the capacity to permeate cell membranes, inducing cell lysis and inflammation. This pathogenic mechanism is pivotal in the etiology of otitis media, pneumonia, meningitis, and bacteremia [57]. Lv et al. have demonstrated that HG exhibits inhibitory effects on PLY’s hemolytic activity and disrupts its oligomerization in a concentration-dependent manner in vitro. Furthermore, HG mitigates PLY-induced cellular damage. Consequently, HG represents a promising lead compound in the development of therapeutic strategies to combat S. pneumoniae infections [58].

**Figure 11:**
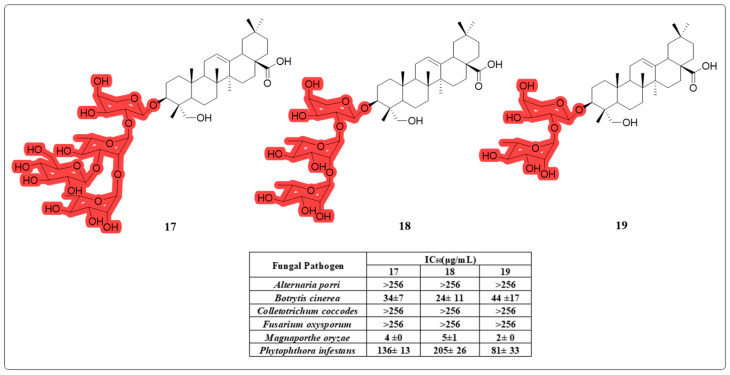
The chemical structures of compounds **17**–**19** as anti-fungal derivatives.

In addition to invading the human body and causing various diseases, fungi also pose a threat to plants. Compared to chemical pesticides, pesticides obtained from natural sources are less toxic to humans and are easier to degrade in natural environments [59,60]. Using activity-controlled fractionation, Choi et al. successfully isolated three antifungal compounds—**17**, **18**, and **19** (Figure 11)—from the methanol extract of *T. palmata*. An in vitro antifungal bioassay demonstrated their potent activities against *M. oryzae*, the causative agent of rice blast. Compound **19** also exhibited significant antifungal activities against tomato and wheat. These findings indicate that HG could serve as a promising source for the development of novel natural fungicides [61].

**Figure 12:**
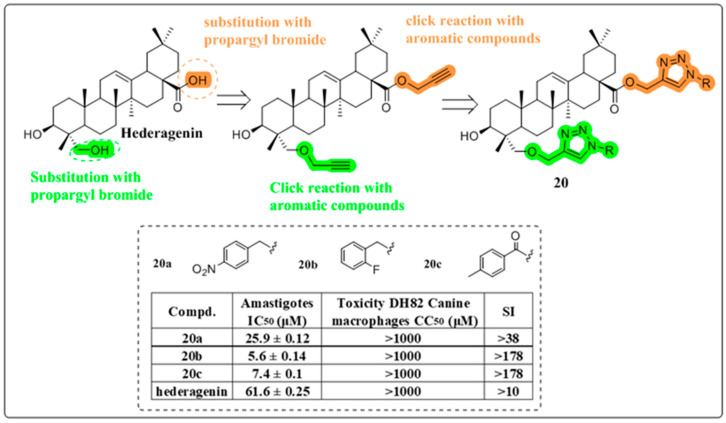
The chemical structure of compound **20** as an anti-leishmania derivative.

Leishmaniasis represents a significant global health challenge caused by protozoan parasites of the genus Leishmania, which are transmitted through the bites of infected female phlebotomine sandflies. This neglected tropical disease manifests in various clinical forms, including cutaneous and visceral leishmaniasis, which can result in severe dermatological manifestations, systemic complications, and potentially fatal outcomes if left untreated [62]. Rene Csuk’s research group aimed to enhance the anti-leishmania activity of HG by synthesizing a series of HG-triazole derivatives covalently linked to C-23 and C-28 positions (Figure 12). Evaluation of the anti-leishmania activity of derivatives **20a** and **20b** revealed remarkable efficacy in vitro. Compound **20c** has more potent selectivity (by 1780-fold) than the commercial antimony drug [63]. Compared to their previous work, the SAR study showed that increasing the number of 1,2,3-triazole fragments could improve the anti-leishmanial activity of HG [19].

### 4.3. Anti-Viral Activity

**Figure 13:**
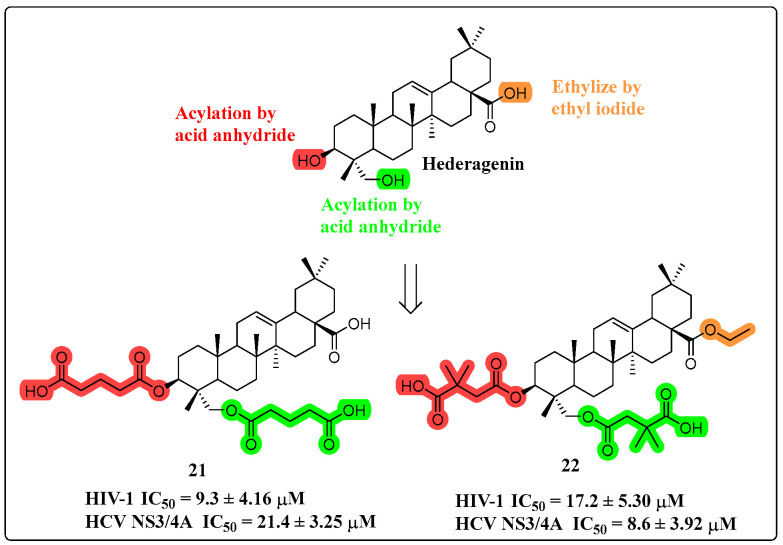
The chemical structures of compounds **21**–**22** as anti-viral derivatives.

There are basically no reports in the literature about the anti-viral activity of HG. However, some reports have confirmed that HG is an inhibitor of NS3/4A proteases of HIV-1 and HCV [64]. In 2022, Liu and colleagues developed a series of HG derivatives through structural modifications at the C-3 and C-23 positions, involving oxidation and acylation reactions (Figure 13). Among the synthesized compounds, derivatives **21** and **22** demonstrated remarkable inhibitory potency against viral NS3/4A proteases, showing IC_50_ values below 10 μM for both HIV-1 and HCV targets. Notably, these compounds exhibited high selectivity, with minimal inhibitory effects on human proteases such as renin and trypsin. SAR analysis revealed that HG-derived dicarboxylic acid half-esters significantly enhanced antiviral activity when C-3 or C-23 was linked to 6-carbon acyl chains [65].

### 4.4. Anti-Fibrosis

Pulmonary fibrosis, a respiratory condition marked by its irreversibility and high morbidity and mortality rates, poses a considerable health risk to humans [66]. Ma et al. systematically investigated the therapeutic potential of HG in bleomycin (BLM)-induced pulmonary dysfunction and associated pathological alterations. Their findings demonstrated that HG treatment significantly reduced hydroxyproline content, suppressed α-SMA expression, and downregulated collagen I production, consequently alleviating extracellular matrix deposition. Furthermore, HG administration markedly decreased the concentrations of pro-inflammatory cytokines in both systemic circulation (serum) and the local pulmonary microenvironment (bronchoalveolar lavage fluid). Further exploration of the mechanisms involved revealed that HG inhibited the expression of Ras, p-JNK and p-NFAT4 [67].

Interstitial fibrosis is the defining pathological feature of chronic kidney disease (CKD) [68]. Jia et al.’s research has demonstrated that HG can significantly ameliorate renal pathological structure and diminish renal fibrosis in a mouse model of CKD. Furthermore, HG demonstrated significant efficacy in downregulating the expression of TGF-β-induced fibrotic markers, including α-SMA and fibronectin FN, in TCMK1 cells. Mechanistic studies revealed a dual role of ISG15; it not only promotes renal tubular epithelial cell fibrosis but also antagonizes the renoprotective effects mediated by HG in CKD. Knocking down ISG15 or increasing the dosage of HG led to a marked inhibition of TGF-β-induced fibrotic protein expression and JAK/STAT pathway activation. Collectively, these findings indicate that HG exerts a potent therapeutic effect on renal fibrosis in CKD, primarily through inhibiting ISG15 and its downstream JAK/STAT signaling pathway [69].

### 4.5. Neuro-Related Activity

Previous research has demonstrated the therapeutic potential of HG in addressing central nervous system (CNS) disorders by enhancing central monoamine signaling. This enhancement occurs via the inhibition of extracellular reuptake of neurotransmitters [27]. Subsequently, An-Guo Wu confirmed HG’s neuroprotective effect through a Parkinson’s disease (PD) mouse model. HG demonstrated significant efficacy in reducing the protein expression of mutant huntingtin (mHTT). Mechanistically, HG was shown to inhibit both α-synuclein oligomerization and the formation of Huntington’s inclusion bodies through activation of AMPK-mTOR-mediated autophagic pathways. In vitro studies further indicated that HG stimulates autophagy and facilitates degradation of neurodegenerative-related proteins, thereby hinting at its potential therapeutic use in neurodegenerative diseases [29].

Lin et al. investigated the protective effects of HG on PC12 cells against corticosterone (CORT)-induced damage. Their results demonstrated a concentration-dependent protective role for HG. Mechanistic investigations revealed that HG effectively attenuated CORT-induced mitochondrial dysfunction by preserving mitochondrial membrane potential (ΔΨm) and reducing ROS accumulation. Furthermore, HG demonstrated significant anti-apoptotic properties against CORT-induced cell death. Importantly, pharmacological inhibition studies using specific PI3K and AKT inhibitors completely abrogated the cytoprotective effects of HG, providing compelling evidence that the PI3K/AKT signaling pathway plays a crucial role in mediating HG’s neuroprotective actions. Additionally, Western blot analysis provided evidence that HG stimulates AKT phosphorylation and the phosphorylation of its downstream targets, such as FoxO3a and GSK3β [70].

Alzheimer’s disease (AD) can induce progressive neurite atrophy and synaptic loss, ultimately leading to neuronal death [71]. The neuroinflammatory microenvironment, predominantly mediated by activated M1-polarized microglia, significantly compromises neurite outgrowth and exacerbates neuronal degeneration. Wang et al. demonstrated that HG effectively attenuates neuroinflammation by inhibiting lipopolysaccharide (LPS)-stimulated NO production and downregulating the expression of key pro-inflammatory cytokines. Furthermore, immunohistochemical analysis of primary microglial cultures revealed that HG treatment significantly reduced the population of Iba-1-positive M1 microglia, suggesting its potential to modulate microglial polarization. HG also significantly inhibited signaling via the activated B cell subunit p65. Furthermore, HG mitigated the neuritic atrophy and neuronal death induced by Aβ25-35. Consequently, HG’s capacity to suppress activated M1 microglia and promote neurite outgrowth may represent a promising therapeutic approach for AD [72].

Stroke represents a significant cause of mortality in China, accounting for 1.2 million deaths annually, predominantly impacting the middle-aged and elderly population [73]. Cerebral ischemia/reperfusion (CI/R) injury is identified as the primary cause of stroke. In an effort to identify effective neuroprotective agents, Yu et al. conducted a study involving mice that were administered HG intraperitoneally for three consecutive days subsequent to middle cerebral artery occlusion (MCAO). The experimental results demonstrated that HG treatment significantly attenuated CI/R-induced neuronal apoptosis and reduced the expression of pro-inflammatory cytokines in the ischemic penumbra. Furthermore, mechanistic investigations revealed that HG exerted its neuroprotective effects through simultaneous inhibition of the MLK3 signaling cascade and downstream MAPK/NF-κB pathways, thereby mitigating CI/R injury. Given HG’s favorable safety profile, it exhibits potential for future clinical application in the treatment of ischemic stroke [74].

### 4.6. Multidrug Resistance Reverse

**Figure 14:**
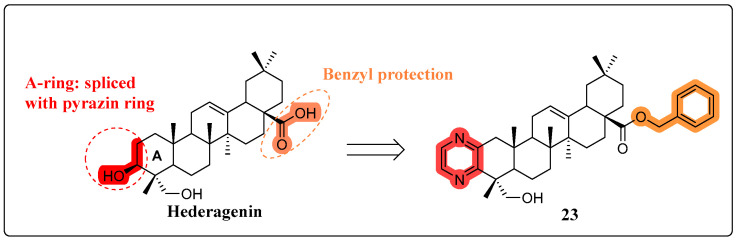
The chemical structure of compound **22** as a multidrug resistance-reversing derivative.

Multidrug resistance (MDR) in cancer treatment poses a formidable challenge, with the overexpression of P-glycoprotein (P-gp) serving as a critical factor [75]. Several natural products have demonstrated the ability to reverse MDR [76,77,78]. In 2018, Yi Bi’s research team synthesized a novel HG derivative, designated as compound **23** (Figure 14), characterized by an A-ring fused to pyrazine. Compound **23** facilitated the accumulation of paclitaxel in MDR cells, without altering the expression levels of P-gp. Moreover, the combination of MDR1-specific siRNA knockdown with compound **23** treatment resulted in a synergistic enhancement of paclitaxel’s cytotoxic efficacy against drug-resistant cancer cells. Importantly, in nude mouse models, compound **23** augmented the antitumor efficacy of paclitaxel against xenograft tumors originating from KBV cancer cells [79].

**Figure 15:**
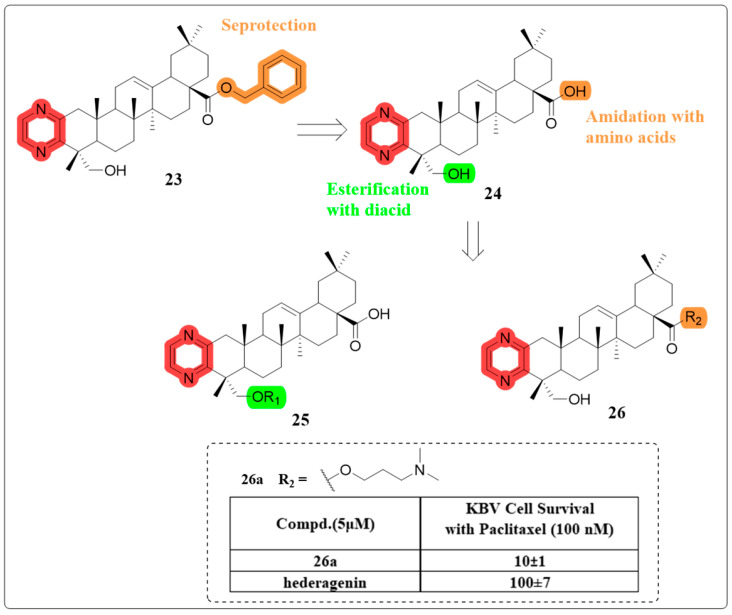
The chemical structures of compounds **24**–**26** as multidrug resistance-reversing derivatives.

In 2019, they successfully synthesized a range of amino derivatives of compound 23, primarily originating from the C-28 carboxyl group (Figure 15). Experimental findings revealed that compound **26a** at a concentration of 5 μM exhibited a marked enhancement in the cytotoxicity of paclitaxel against several MDR cells. Additionally, compound **26a** was observed to inhibit the drug efflux of P-gp by stimulating its ATPase activity. Compound **26a** augmented paclitaxel’s efficacy in xenograft tumors derived from KBV cancer cells [80].

**Figure 16:**
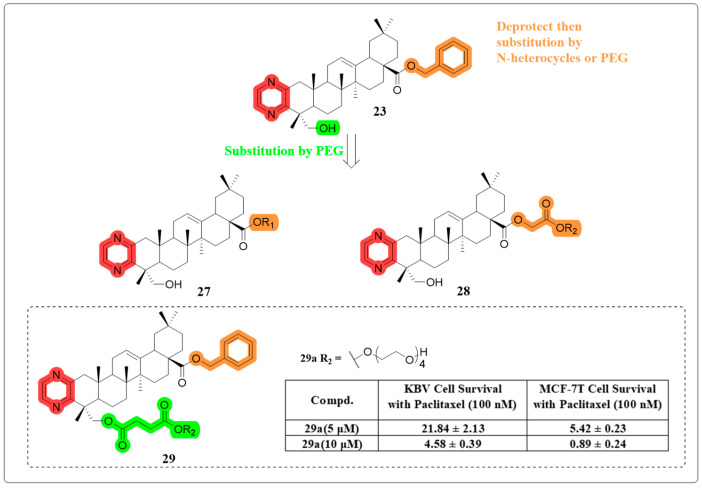
The chemical structures of compounds **27**–**29** as multidrug resistance-reversing derivatives.

In 2021, they synthesized three series of derivatives designed to enhance the water solubility and reverse cancer MDR activity of compound **23** (Figure 16). Specifically, in MTT assays performed on KBV cells that were treated with paclitaxel at a concentration of 10 mM, these derivatives effectively reversed the MDR phenotype. Among them, the PEGylated derivative **29a** stood out, exhibiting a 20-fold increase in water solubility compared to compound **23**, while maintaining its tumor MDR reversal activity [81].

**Figure 17:**
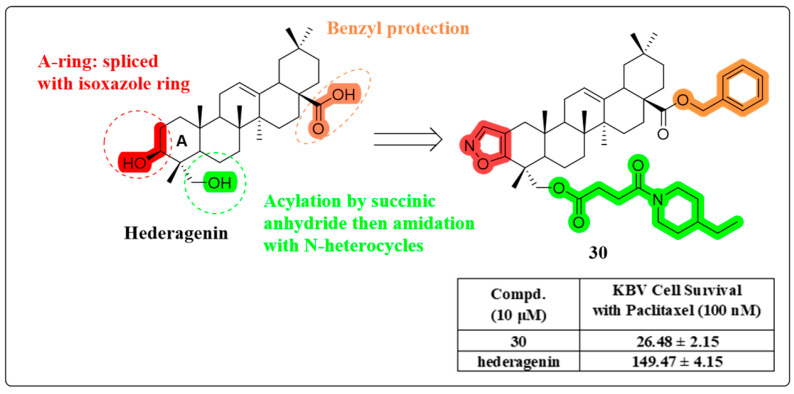
The chemical structure of compound **30** as a multidrug resistance-reversing derivative.

In 2022, three series of HG derivatives were successfully synthesized through the strategic utilization of A-ring fusion and the innovative inclusion of nitrogen-containing heterocycles, along with benzyl group substitution. Compound **30** (Figure 17) demonstrated a significantly enhanced capacity to reverse multidrug resistance (MDR), exhibiting a mechanism akin to that of compound **23**. Notably, compound **30** demonstrated significant synergistic effects with paclitaxel in a KBV cell-derived xenograft mouse model, achieving a remarkable tumor growth inhibition rate of 56.24%. This represents a substantial 30% enhancement in therapeutic efficacy compared to previous treatment regimens. These findings underscore the pivotal role of nitrogen-containing heterocycles as a structural fragment within HG MDR reversal derivatives [82].

### 4.7. Anti-Inflammatory Activity

Alcoholic liver disease is marked by lipid accumulation, inflammation, and apoptosis, ultimately progressing to cirrhosis, fibrosis, and hepatocellular carcinoma [83]. Gyeong-Ji Kim et al. investigated the effects of HG in rats with alcohol-induced hepatotoxicity and observed an upregulation of acetaldehyde dehydrogenase-2 mRNA expression following HG treatment. HG mitigated the elevation of pro-inflammatory cytokines. Western blot analysis further revealed that HG increased Bcl-2 expression while decreasing the levels of Bax and p53. HG treatment attenuated the activation of p38 MAPK induced by ethanol and enhanced the phosphorylation of AKT and ERK [84].

Li et al. investigated the therapeutic potential of HG in diabetic cardiomyopathy, focusing on its impact on cardiac pathology and heart failure. The study revealed that treatment with HG led to a reduction in pro-inflammatory cytokine levels and decreased both heart mass and body weight in diabetic db/db mice, without affecting their fasting plasma glucose (FPG) levels. HG proved effective in ameliorating cardiac dysfunction, myocardial hypertrophy, and fibrosis in these animals. Additionally, HG inhibited the NF-κB and Smad signaling pathways, leading to decreased attack in the diabetic hearts. These findings suggest that the cardioprotective effects of HG may be mediated through the suppression of inflammatory-related NF-κB and Smad signaling pathways [85].

Acute lung injury (ALI) stands as a prominent cause of mortality in sepsis patients [86]. Wang et al. exhibited that the administration of HG resulted in improved survival rates, mitigated lung injury, and reduced both the wet-to-dry ratio in the lungs and the accumulation of inflammatory cells in the bronchoalveolar lavage fluid (BALF) of rats with ALI. HG treatment resulted in a reduction of iNOS, COX-2, and CD86 expression levels within lung tissue, along with decreased secretion of proinflammatory cytokines. Furthermore, it inhibited the polarization of M1 macrophages and curbed the production of M1-associated proinflammatory mediators. Mechanistically, HG suppressed the activation of the NLRP3 inflammasome and NF-κB pathway [87].

**Figure 18:**
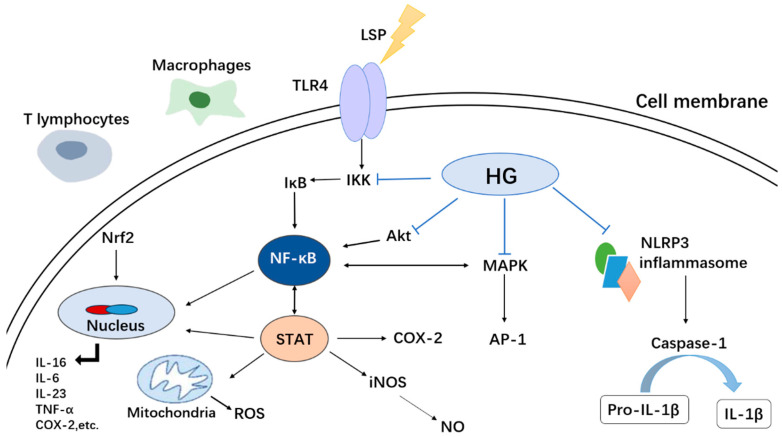
Mechanism underlying the anti-inflammatory activity of HG.

Systemic inflammatory responses often lead to sepsis, necessitating the development of effective anti-inflammatory strategies for its management [88]. Yu et al. synthesized thirty-two HG derivatives, incorporating modifications at specific positions—the A-ring, C-28, and C-23—to evaluate their anti-inflammatory effects in vitro. Notably, compound **24** (Figure 18) demonstrated the highest potency, effectively inhibiting related proteins within the cGAS-STING signaling pathway. These results suggest that compound **24** attenuates inflammation by suppressing STING expression, thereby modulating both STING and NF-κB signaling cascades. In a mouse model of sepsis accompanied by acute LPS-induced liver injury, compound **24** exhibited a significant reduction in inflammatory responses [89].

### 4.8. Osteoclast Inhibition Activity

Enhanced osteoclastogenesis and osteoclast-mediated bone resorption can lead to significant bone loss [90]. Kun Tian’s research revealed that HG acts by suppressing RANKL-induced intracellular ROS production and activation of the MAPK pathway, subsequently inhibiting the downstream expression of c-Fos and NFATc1. HG has the capacity to inhibit bone resorption and osteoclast formation via RANKL. In vivo studies utilizing an ovariectomy (OVX)-induced bone loss mouse model have shown that HG effectively alleviates bone loss in OVX mice by reducing both the quantity and functional activity of osteoclasts on bone surfaces [91]. These intriguing and promising findings suggest that HG may have potential as a therapeutic option for the treatment of osteolytic bone loss.

### 4.9. Renal Disease

Xie et al. conducted transcriptome sequencing to investigate the role of LncRNA-A33 in acute kidney injury (AKI) [92]. Their findings revealed that LncRNA-A33 was markedly upregulated in the kidneys of AKI mice, as well as in LPS-induced renal tubule cells. Subsequently, through the use of an in situ kidney electroporation technique to knockdown the A33 plasmid in AKI mouse kidneys, it was observed that inhibiting A33 significantly mitigated cisplatin-induced renal damage and inflammation. The results demonstrated a downregulation of numerous inflammation-related signaling pathways, with particular inhibition noted in the Axin2 and its downstream β-catenin signaling pathway. Furthermore, a cell recovery assay indicated that high glucose (HG) inhibited the Axin2/β-catenin signaling by downregulating A33, thereby ameliorating AKI-associated renal damage and inflammation [93].

Diabetic nephropathy (DN) is a common complication of diabetes which is distinguished by renal fibrosis and a grim prognosis for affected patients [94]. Jia et al. uncovered that HG can suppress ferroptosis and the subsequent fibrosis triggered by erastin, a known ferroptosis inducer, in renal tubule cells. Mechanistic investigations have revealed that Smad3 phosphorylation promotes ferroptosis in these cells, ultimately contributing to renal fibrosis. Conversely, HG functions to inhibit Smad3 phosphorylation, resulting in an upregulation of glutathione peroxidase 4 (GPX4). This upregulation, in turn, mitigates ferroptosis-induced renal fibrosis [95]. These observations indicate that HG, as a naturally occurring substance, possesses therapeutic promise for alleviating renal fibrosis associated with DN.

## 5. Conclusions

This review represents a timely addition to the previous review of the structural variation and biological activity of HG. Over the last decade, interest in HG has increased. Researchers have endeavored to introduce various structural modifications to HG. Through the assessment of the activity of the resultant derivatives, they have uncovered several crucial structure–activity relationships, which could facilitate the rapid identification of more potent derivatives. With the development of new technologies and experimental methods, great progress has been made in expanding the pharmacological activities and mechanisms of HG in vitro and in vivo. Several new activities, mechanisms and target proteins were found, proving the value of HG in disease therapy, healthcare and crop protection.

Although there have been many advances in HG research in the last decade, there are still some pressing issues that need to be resolved. HG has relatively low bioavailability because it is poorly water soluble, which limits the application of HG in clinical applications. This problem has been around for a long time and there is limited research on it. Current research on HG primarily centers on its pharmacology and structural modification, with relatively limited studies on pharmacokinetics and toxicology.

To advance HG as a viable therapeutic option, novel research directions in these areas are necessary for future exploration. First, the bioavailability of HG could be increased through appropriate pharmaceutical techniques and modification methods. For example, improving solubility through the use of inclusions, nanopreparations or solid dispersions increases bioavailability. Second, to validate the efficacy of HG, sufficient pharmacokinetic and toxicological studies must be performed. Finally, many studies of HG derivatives have focused and continue to focus on anti-cancer effects. However, the various studies on other biological activities are not in-depth enough. To fully harness the bioactivities of HG, conducting systematic derivatization studies across various HG-related diseases could lead to the discovery of more favorable molecules with enhanced biological profiles.

## References

[1] Li G., Lou H.X. (2018). Strategies to diversify natural products for drug discovery. Med. Res. Rev..

[2] Naaz F., Haider M.R., Shafi S., Yar M.S. (2019). Anti-tubulin agents of natural origin: Targeting taxol, vinca, and colchicine binding domains. Eur. J. Med. Chem..

[3] Zhang N., Xu W., Yan Y.S., Chen M.J., Li H., Chen L.X. (2023). Cembrane diterpenoids: Chemistry and pharmacological activities. Phytochemistry.

[4] Farco J.A., Grundmann O. (2013). Menthol—Pharmacology of an Important Naturally Medicinal “Cool”. Mini-Rev. Med. Chem..

[5] Salvador J.A.R., Leal A.S., Valdeira A.S., Goncalves B.M.F., Alho D.P.S., Figueiredo S.A.C., Silvestre S.M., Mendes V.I.S. (2017). Oleanane-, ursane-, and quinone methide friedelane-type triterpenoid derivatives: Recent advances in cancer treatment. Eur. J. Med. Chem..

[6] Huang J., Wang Y.H., Li C., Wang X.L., He X.J. (2016). Triterpenes isolated from acorns of Quercus serrata var. brevipetiolata exert anti-inflammatory activity. Ind. Crops Prod..

[7] Xiao S., Tian Z., Wang Y., Si L., Zhang L., Zhou D. (2018). Recent progress in the antiviral activity and mechanism study of pentacyclic triterpenoids and their derivatives. Med. Res. Rev..

[8] Mabhida S.E., Dludla P.V., Johnson R., Ndlovu M., Louw J., Opoku A.R., Mosa R.A. (2018). Protective effect of triterpenes against diabetes-induced beta-cell damage: An overview of in vitro and in vivo studies. Pharmacol. Res..

[9] Popova M.P., Chinou I.B., Marekov I.N., Bankova V.S. (2009). Terpenes with antimicrobial activity from Cretan propolis. Phytochemistry.

[10] Sanchez-Quesada C., Lopez-Biedma A., Warleta F., Campos M., Beltran G., Gaforio J.J. (2013). Bioactive Properties of the Main Triterpenes Found in Olives, Virgin Olive Oil, and Leaves of Olea europaea. J. Agric. Food. Chem..

[11] Morikawa T., Ninomiya K., Imura K., Yamaguchi T., Akagi Y., Yoshikawa M., Hayakawa T., Muraoka O. (2014). Hepatoprotective triterpenes from traditional Tibetan medicine Potentilla anserina. Phytochemistry.

[12] Sanchez M., Theoduloz C., Schmeda-Hitschmann G., Razmilic I., Yanez T., Rodriguez J.A. (2006). Gastroprotective and ulcer-healing activity of oleanolic acid derivatives: In vitro-in vivo relationships. Life Sci..

[13] Ou-Yang Q., Xuan C.X., Wang X., Luo H.Q., Liu J.E., Wang L.L., Li T.T., Chen Y.P., Liu J. (2018). 3-Acetyl-oleanolic acid ameliorates non-alcoholic fatty liver disease in high fat diet-treated rats by activating AMPK-related pathways. Acta Pharmacol. Sin..

[14] Yang C.K., Pan Q.Q., Ji K., Tian Z., Zhou H.Y., Li S.H., Luo C.C., Li J. (2023). Review on the protective mechanism of astragaloside IV against cardiovascular diseases. Front. Pharmacol..

[15] Yin M.C., Chan K.C. (2007). Nonenzymatic antioxidative and antiglycative effects of oleanolic acid and ursolic acid. J. Agric. Food. Chem..

[16] Chen Y.Y., Liu Q.P., An P., Jia M., Luan X., Tang J.Y., Zhang H. (2022). Ginsenoside Rd: A promising natural neuroprotective agent. Phytomedicine.

[17] Woldemichael G.M., Wink M. (2001). Identification and biological activities of triterpenoid saponins from Chenopodium quinoa. J. Agric. Food. Chem..

[18] Lee C.W., Park S.M., Zhao R., Lee C., Chun W., Son Y., Kim S.H., Jung J.Y., Jegal K.H., Cho I.J. (2015). Hederagenin, a major component of Clematis mandshurica Ruprecht root, attenuates inflammatory responses in RAW 264.7 cells and in mice. Int. Immunopharmacol..

[19] Rodriguez-Hernandez D., Barbosa L.C.A., Demuner A.J., de Almeida R.M., Fujiwara R.T., Ferreira S.R. (2016). Highly potent anti-leishmanial derivatives of hederagenin, a triperpenoid from *Sapindus saponaria* L. Eur. J. Med. Chem..

[20] Luo J.-G., Liu J., Kong L.-Y. (2008). New pentacyclic triterpenes from Gypsophila oldhamiana and their biological evaluation as glycogen phosphorylase inhibitors. Chem. Biodivers..

[21] Jin Z.-L., Gao N., Zhou D., Chi M.-G., Yang X.-M., Xu J.-P. (2012). The extracts of Fructus Akebiae, a preparation containing 90% of the active ingredient hederagenin: Serotonin, norepinephrine and dopamine reuptake inhibitor. Pharmacol. Biochem. Behav..

[22] Zeng J., Huang T., Xue M., Chen J.X., Feng L.L., Du R.F., Feng Y. (2018). Current knowledge and development of hederagenin as a promising medicinal agent: A comprehensive review. RSC Adv..

[23] Yang X.M., Li G.L., Chen L.Y., Zhang C., Wan X.X., Xu J.P. (2011). Quantitative determination of hederagenin in rat plasma and cerebrospinal fluid by ultra fast liquid chromatography-tandem mass spectrometry method. J. Chromatogr. B-Anal. Technol. Biomed. Life Sci..

[24] Zhang R., Zhu H., Ding L., Yang Z.L. (2014). Determination of asperosaponin VI and its active metabolite hederagenin in rat tissues by LC-MS/MS: Application to a tissue distribution study. J. Chromatogr. B-Anal. Technol. Biomed. Life Sci..

[25] Wang W., Wu Y., Yang K., Wu C., Tang R., Li H., Chen L. (2019). Synthesis of novel andrographolide beckmann rearrangement derivatives and evaluation of their HK2-related anti-inflammatory activities. Eur. J. Med. Chem..

[26] Oh S.R., Jung K.Y., Son K.H., Park S.H., Lee I.S., Ahn K.S., Lee H.K. (1999). In vitro anticomplementary activity of hederagenin saponins isolated from roots of Dipsacus asper. Arch. Pharmacal Res..

[27] Zhou D., Jin H., Lin H.-B., Yang X.-M., Cheng Y.-F., Deng F.-J., Xu J.-P. (2010). Antidepressant effect of the extracts from Fructus Akebiae. Pharmacol. Biochem. Behav..

[28] Liang B.-F., Huang F., Wang H.-T., Wang G.-H., Yuan X., Zhang M.-Z., Guo H.-B., Cheng Y.-F., Xu J.-P. (2015). Involvement of norepinephrine and serotonin system in antidepressant-like effects of hederagenin in the rat model of unpredictable chronic mild stress-induced depression. Pharm. Biol..

[29] Wu A.-G., Zeng W., Wong V.K.-W., Zhu Y.-Z., Lo A.C.Y., Liu L., Law B.Y.-K. (2017). Hederagenin and α-hederin promote degradation of proteins in neurodegenerative diseases and improve motor deficits in MPTP-mice. Pharmacol. Res..

[30] Wu A.-G., Wong V.K.-W., Zeng W., Liu L., Law B.Y.-K. (2015). Identification of novel autophagic *Radix Polygalae* fraction by cell membrane chromatography and UHPLC-(Q) TOF-MS for degradation of neurodegenerative disease proteins. Sci. Rep..

[31] Lu S.-H., Guan J.-H., Huang Y.-L., Pan Y.-W., Yang W., Lan H., Huang S., Hu J., Zhao G.-P. (2015). Experimental Study of Antiatherosclerosis Effects with Hederagenin in Rats. Evid. Based Complement. Alternat. Med..

[32] Park H.J., Kim D.H., Choi J.W., Park J.H., Han Y.N. (1998). A potent anti-diabetic agent from Kalopanax pictus. Arch. Pharmacal Res..

[33] Liu B.-X.-Z., Zhou J.-Y., Li Y., Zou X., Wu J., Gu J.-F., Yuan J.-R., Zhao B.-J., Feng L., Jia X.-B. (2014). Hederagenin from the leaves of ivy (*Hedera helix* L.) induces apoptosis in human LoVo colon cells through the mitochondrial pathway. BMC Complement. Altern. Med..

[34] Gao Y., He C., Bi W., Wu G., Altman E. (2016). Bioassay Guided Fractionation Identified Hederagenin as a Major Cytotoxic Agent from Cyclocarya paliurus Leaves. Planta Med..

[35] Wu X., Gao H., Hou Y., Yu J., Sun W., Wang Y., Chen X., Feng Y., Xu Q.-m., Chen X. (2018). Dihydronortanshinone, a natural product, alleviates LPS-induced inflammatory response through NF-κB, mitochondrial ROS, and MAPK pathways. Toxicol. Appl. Pharmacol..

[36] Ziech D., Anestopoulos I., Hanafi R., Voulgaridou G.P., Franco R., Georgakilas A.G., Pappa A., Panayiotidis M.I. (2012). Pleiotrophic effects of natural products in ROS-induced carcinogenesis: The role of plant-derived natural products in oral cancer chemoprevention. Cancer Lett..

[37] Ke Y., Wang W., Zhao L.-F., Liang J.-J., Liu Y., Zhang X., Feng K., Liu H.-M. (2018). Design, synthesis and biological mechanisms research on 1,2,3-triazole derivatives of Jiyuan Oridonin A. Biorg. Med. Chem..

[38] Kim E.H., Baek S., Shin D., Lee J., Roh J.L. (2017). Hederagenin Induces Apoptosis in Cisplatin-Resistant Head and Neck Cancer Cells by Inhibiting the Nrf2-ARE Antioxidant Pathway. Oxidative Med. Cell. Longev..

[39] Wang K., Liu X.D., Liu Q.M., Ho I.H., Wei X.L., Yin T., Zhan Y.J., Zhang W.J., Zhang W.B., Chen B.N. (2020). Hederagenin potentiated cisplatin- and paclitaxel-mediated cytotoxicity by impairing autophagy in lung cancer cells. Cell Death Dis..

[40] Diwanji N., Bergmann A. (2018). An unexpected friend—ROS in apoptosis-induced compensatory proliferation: Implications for regeneration and cancer. Semin. Cell Dev. Biol..

[41] Chen H., Guan X., Liu Q., Yang L., Guo J., Gao F., Qi Y., Wu X., Zhang F., Tian X. (2022). Co-assembled Nanocarriers of *De Novo* Thiol-Activated Hydrogen Sulfide Donors with an RGDFF Pentapeptide for Targeted Therapy of Non-Small-Cell Lung Cancer. ACS Appl. Mater. Interfaces.

[42] Liu Z., Tan X.N., Peng L., Gao W.H., Zeng P.H. (2024). Hederagenin Induces Apoptosis of Human Hepatoma HepG2 Cells via the Mitochondrial Pathway. Comb. Chem. High. Throughput Screen..

[43] Dai Y.X., Masra N., Zhou L., Yu C., Jin W., Ni H.B. (2023). Hederagenin suppresses glioma cell biological activities via Nur77 in vitro study. Food Sci. Nutr..

[44] Wu Y., Yang Y., Wang W., Sun D., Liang J., Zhu M., Li H., Chen L. (2022). PROTAC technology as a novel tool to identify the target of lathyrane diterpenoids. Acta Pharm. Sin. B.

[45] Zhang Y.S., Han Y., Shang Y.C., Wang X.Y., Sun J.W. (2023). Proteomics identifies differentially expressed proteins in glioblastoma U87 cells treated with hederagenin. Proteome Sci..

[46] Su F., Sui X., Xu J.B., Liu Q.L., Li J.F., Liu W.H., Xu Y., Zhang Z.Q., Tao F.F. (2024). Hederagenin suppresses ovarian cancer via targeting mitochondrial fission through dynamin-related protein 1. Eur. J. Pharmacol..

[47] Hu S., Chu Y., Zhou X., Wang X. (2023). Recent advances of ferroptosis in tumor: From biological function to clinical application. Biomed. Pharmacother..

[48] Lu J.Y., Guo Q.X., Zhao H., Liu H. (2024). Hederagenin promotes lung cancer cell death by activating CHAC1-dependent ferroptosis pathway. Biochem. Biophys. Res. Commun..

[49] Cheng L., Shi L., Wu J., Zhou X.J., Li X.X., Sun X., Zhu L., Xia T.S., Ding Q. (2018). A hederagenin saponin isolated from Clematis ganpiniana induces apoptosis in breast cancer cells via the mitochondrial pathway. Oncol. Lett..

[50] Liu X.X., Yang Y.T., Wang X., Wang K.Y., Liu J.Q., Lei L., Luo X.M., Zhai R., Fu F.H., Wang H.B. (2017). Design, synthesis and biological evaluation of novel alpha-hederagenin derivatives with anticancer activity. Eur. J. Med. Chem..

[51] Chen Z., Duan H., Wang M., Han L., Liu Y., Zhu Y., Yang S. (2015). Synthesis, cytotoxicity and haemolytic activity of Pulsatilla saponin A, D derivatives. Bioorg. Med. Chem. Lett..

[52] Chen Z., Duan H.Q., Tong X.H., Hsu P.L., Han L., Morris-Natschke S.L., Yang S.L., Liu W., Lee K.H. (2018). Cytotoxicity, Hemolytic Toxicity, and Mechanism of Action of Pulsatilla Saponin D and Its Synthetic Derivatives. J. Nat. Prod..

[53] Fu X., Lu H., Gao M., Li P., He Y., He Y., Luo X., Rao X., Liu W. (2024). Nitric oxide in the cardio-cerebrovascular system: Source, regulation and application. Nitric Oxide-Biol. Chem..

[54] Chen Z., Huang K.Y., Ling Y., Goto M., Duan H.Q., Tong X.H., Liu Y.L., Cheng Y.Y., Morris-Natschke S.L., Yang P.C. (2019). Discovery of an Oleanolic Acid/Hederagenin-Nitric Oxide Donor Hybrid as an EGFR Tyrosine Kinase Inhibitor for Non-Small-Cell Lung Cancer. J. Nat. Prod..

[55] Rodriguez-Hernandez D., Barbosa L.C.A., Demuner A.J., Martins J.P.A., Fischer L., Csuk R. (2019). Hederagenin amide derivatives as potential antiproliferative agents. Eur. J. Med. Chem..

[56] Fang K., Zhang X.H., Han Y.T., Wu G.R., Cai D.S., Xue N.N., Guo W.B., Yang Y.Q., Chen M., Zhang X.Y. (2018). Design, Synthesis, and Cytotoxic Analysis of Novel Hederagenin-Pyrazine Derivatives Based on Partial Least Squares Discriminant Analysis. Int. J. Mol. Sci..

[57] Anderson R., Feldman C. (2017). Pneumolysin as a potential therapeutic target in severe pneumococcal disease. J. Infect..

[58] Ding R., Zhang Y., Xu X.Z., Hou Y.F., Nie J., Deng X.M., Qiu J.Z., Lv Q.H. (2022). Inhibitory effect of hederagenin on Streptococcus pneumoniae pneumolysin in vitro. Microb. Infect..

[59] Xu M., Xu J., Hao M., Zhang K., Lv M., Xu H. (2019). Evaluation of andrographolide-based analogs derived from Andrographis paniculata against Mythimna separata Walker and Tetranychus cinnabarinus Boisduval. Bioorg. Chem..

[60] Huang X., Zhang B., Xu H. (2018). Synthesis of andrographolide-related esters as insecticidal and acaricidal agents. Bioorg. Med. Chem. Lett..

[61] Kim B., Han J.W., Ngo M.T., Quang Le D., Kim J.-C., Kim H., Choi G.J. (2018). Identification of novel compounds, oleanane- and ursane-type triterpene glycosides, from Trevesia palmata: Their biocontrol activity against phytopathogenic fungi. Sci. Rep..

[62] Coffeng L.E., de Vlas S.J., Singh R.P., James A., Bindroo J., Sharma N.K., Ali A., Singh C., Sharma S., Coleman M. (2024). Effect of indoor residual spraying on sandfly abundance and incidence of visceral leishmaniasis in India, 2016–2022: An interrupted time-series analysis and modelling study. Lancet. Infect. Dis..

[63] Rodriguez-Hernandez D., Barbosa L.C.A., Demuner A.J., Nain-Perez A., Ferreira S.R., Fujiwara R.T., de Almeida R.M., Heller L., Csuk R. (2017). Leishmanicidal and cytotoxic activity of hederagenin-bistriazolyl derivatives. Eur. J. Med. Chem..

[64] Wei Y., Ma C.M., Chen D.Y., Hattori M. (2008). Anti-HIV-1 protease triterpenoids from Stauntonia obovatifoliola Hayata subsp intermedia. Phytochemistry.

[65] Liu Q., Wei Y., Hao Y., Yang J., Pan B., Yang X., Zhou Y., Wang X. (2022). Synthesis and Evaluation of Acylated Derivatives of Hederagenin as Inhibitors of HIV-1 and HCV NS3/4A Proteases. Nat. Prod. Commun..

[66] Moss B.J., Ryter S.W., Rosas I.O. (2022). Pathogenic Mechanisms Underlying Idiopathic Pulmonary Fibrosis. Annu. Rev. Pathol. Mech. Dis..

[67] Ma W.J., Huang Q.S., Xiong G.F., Deng L.J., He Y. (2020). The protective effect of Hederagenin on pulmonary fibrosis by regulating the Ras/JNK/NFAT4 axis in rats. Biosci. Biotechnol. Biochem..

[68] Wang Y.Y., Jiang H., Pan J., Huang X.R., Wang Y.C., Huang H.F., To K.F., Nikolic-Paterson D.J., Lan H.Y., Chen J.H. (2017). Macrophage-to-Myofibroblast Transition Contributes to Interstitial Fibrosis in Chronic Renal Allograft Injury. J. Am. Soc. Nephrol..

[69] Jia J., Xu L.H., Deng C., Zhong X., Xie K.H., Han R.Y., Su H.W., Tan R.Z., Wang L. (2023). Hederagenin ameliorates renal fibrosis in chronic kidney disease through blocking ISG15 regulated JAK/STAT signaling. Int. Immunopharmacol..

[70] Lin R.H., Liu L.L., Silva M., Fang J.K., Zhou Z.W., Wang H.T., Xu J.P., Li T.J., Zheng W.H. (2021). Hederagenin Protects PC12 Cells Against Corticosterone-Induced Injury by the Activation of the PI3K/AKT Pathway. Front. Pharmacol..

[71] Scheltens P., De Strooper B., Kivipelto M., Holstege H., Chetelat G., Teunissen C.E., Cummings J., van der Flier W.M. (2021). Alzheimer’s disease. Lancet.

[72] Wang H., Zhang C., Yang L.-E., Yang Z. (2020). Hederagenin Modulates M1 Microglial Inflammatory Responses and Neurite Outgrowth. Nat. Prod. Commun..

[73] Kargiotis O., Safouris A., Psychogios K., Saposnik G., Yaghi S., Merkler A., Kamel H., Filippatos G., Tsivgoulis G. (2024). Heart failure and stroke: The underrepresentation of the heart failure with preserved ejection fraction subtype in randomized clinical trials of therapeutic anticoagulation. J. Neurol. Sci..

[74] Yu H.L., Song L.L., Cao X., Li W., Zhao Y.Y., Chen J., Li J., Chen Y.Z., Yu W.K., Xu Y. (2020). Hederagenin Attenuates Cerebral Ischaemia/Reperfusion Injury by Regulating MLK3 Signalling. Front. Pharmacol..

[75] Chen T., Xiao Z., Liu X., Wang T., Wang Y., Ye F., Su J., Yao X., Xiong L., Yang D.-H. (2024). Natural products for combating multidrug resistance in cancer. Pharmacol. Res..

[76] Xia Y.-Z., Ni K., Guo C., Zhang C., Geng Y.-D., Wang Z.-D., Yang L., Kong L.-Y. (2015). Alopecurone B reverses doxorubicin-resistant human osteosarcoma cell line by inhibiting P-glycoprotein and NF-kappa B signaling. Phytomedicine.

[77] Chang Y.-T., Wang C.C.N., Wang J.-Y., Lee T.-E., Cheng Y.-Y., Morris-Natschke S.L., Lee K.-H., Hung C.-C. (2019). Tenulin and isotenulin inhibit P-glycoprotein function and overcome multidrug resistance in cancer cells. Phytomedicine.

[78] Hsiao S.-H., Lu Y.-J., Yang C.-C., Tuo W.-C., Lo Y.-Q., Huang Y.-H., Hsieh C.-H., Hung T.-H., Wu C.-P. (2016). Hernandezine, a Bisbenzylisoquinoline Alkaloid with Selective Inhibitory Activity against Multidrug-Resistance-Linked ATP-Binding Cassette Drug Transporter ABCB1. J. Nat. Prod..

[79] Yang Y.T., Guan D.K., Lei L., Lu J., Liu J.Q., Yang G.Q., Yan C.H., Zhai R., Tian J.W., Bi Y. (2018). H6, a novel hederagenin derivative, reverses multidrug resistance in vitro and in vivo. Toxicol. Appl. Pharmacol..

[80] Wang X., Ren Q.W., Liu X.X., Yang Y.T., Wang B.H., Zhai R., Qi J.G., Tian J.W., Wang H.B., Bi Y. (2019). Synthesis and biological evaluation of novel H6 analogues as drug resistance reversal agents. Eur. J. Med. Chem..

[81] Wang B., Liu S., Huang W., Ma M., Chen X., Zeng W., Liang K., Wang H., Bi Y., Li X. (2021). Design, synthesis, and biological evaluation of hederagenin derivatives with improved aqueous solubility and tumor resistance reversal activity. Eur. J. Med. Chem..

[82] Huang W., Wang Y., Xu S., Qiao H., Cheng H., Wang L., Liu S., Tian Q., Wang R., Wang H. (2022). Design, synthesis, and tumor drug resistance reversal activity of novel hederagenin derivatives modified by nitrogen-containing heterocycles. Eur. J. Med. Chem..

[83] Duryee M.J., Aripova N., Hunter C.D., Ruskamp R.J., Tessin M.R., Works D.R., Mikuls T.R., Thiele G.M. (2022). A novel reactive aldehyde species inhibitor prevents the deleterious effects of ethanol in an animal model of alcoholic liver disease. Int. Immunopharmacol..

[84] Kim G.-J., Song D.H., Yoo H.S., Chung K.-H., Lee K.J., An J.H. (2017). Hederagenin Supplementation Alleviates the Pro-Inflammatory and Apoptotic Response to Alcohol in Rats. Nutrients.

[85] Li Y., Dong J., Shang Y., Zhao Q., Li P., Wu B. (2019). Anti-inflammatory effects of hederagenin on diabetic cardiomyopathy via inhibiting NF-kappa B and Smads signaling pathways in a type-2 diabetic mice model. RSC Adv..

[86] Zhang Z., Yang X.X., Meng Q.H., Long Y.Y., Shi X.F., Wang Y.L. (2023). Adipose tissue-derived mesenchymal stromal cells attenuate acute lung injury induced by trauma and haemorrhagic shock. Immunobiology.

[87] Wang L., Zhao M. (2022). Suppression of NOD-like receptor protein 3 inflammasome activation and macrophage M1 polarization by hederagenin contributes to attenuation of sepsis-induced acute lung injury in rats. Bioengineered.

[88] Sun Y., Sun S.J., Chen P., Dai Y., Yang D., Lin Y., Yi L.S. (2024). Maresins as novel anti-inflammatory actors and putative therapeutic targets in sepsis. Pharmacol. Res..

[89] Yu T., Cheng H.R., Li X.L., Huang W.T., Li H.X., Gao X.J., Zhao J.N., Zhang X., Gu X.X., Bi Y. (2023). Design and synthesis of hederagenin derivatives modulating STING/NF-?B signaling for the relief of acute liver injury in septic mice. Eur. J. Med. Chem..

[90] Pimenta-Lopes C., Sanchez-de-Diego C., Deber A., Egea-Cortes A., Valer J.A., Alcala A., Mendez-Lucas A., Esteve-Codina A., Rosa J.L., Ventura F. (2023). Inhibition of C5AR1 impairs osteoclast mobilization and prevents bone loss. Mol. Ther..

[91] Tian K., Su Y., Ding J., Wang D., Zhan Y., Li Y., Liang J., Lin X., Song F., Wang Z. (2020). Hederagenin protects mice against ovariectomy-induced bone loss by inhibiting RANKL-induced osteoclastogenesis and bone resorption. Life Sci..

[92] Atak M., Yigit E., Huner Yigit M., Topal Suzan Z., Yilmaz Kutlu E., Karabulut S. (2024). Synthetic and non-synthetic inhibition of ADAM10 and ADAM17 reduces inflammation and oxidative stress in LPS-induced acute kidney injury in male and female mice. Eur. J. Pharmacol..

[93] Xie K.H., Liu X.H., Jia J., Zhong X., Han R.Y., Tan R.Z., Wang L. (2022). Hederagenin ameliorates cisplatin-induced acute kidney injury via inhibiting long non-coding RNA A330074k22Rik/Axin2/13-catenin signalling pathway. Int. Immunopharmacol..

[94] ElKhooly I.A., El-Bassossy H.M., Mohammed H.O., Atwa A.M., Hassan N.A. (2024). Vitamin B1 and calcitriol enhance glibenclamide suppression of diabetic nephropathy: Role of HMGB1/TLR4/NF-kappaB/TNF-alpha/Nrf2/alpha-SMA trajectories. Life Sci..

[95] Jia J., Tan R.Z., Xu L.H., Wang H.L., Li J.C., Su H.W., Zhong X., Liu P., Wang L. (2024). Hederagenin improves renal fibrosis in diabetic nephropathy by regulating Smad3/NOX4/SLC7A11 signaling-mediated tubular cell ferroptosis. Int. Immunopharmacol..

